# A Rapid Systematic Review on the Experiences of Cancer Survivors Residing in Rural Areas during the COVID-19 Pandemic

**DOI:** 10.3390/ijerph192416863

**Published:** 2022-12-15

**Authors:** David Nelson, Samuel Cooke, Ben McLeod, Agnes Nanyonjo, Ros Kane, Mark Gussy

**Affiliations:** 1 Lincoln International Institute for Rural Health, University of Lincoln, Brayford Pool, Lincoln LN6 7TS, UK; 2 Macmillan Cancer Support, London SE1 7UQ, UK; 3 School of Health and Social Care, College of Social Science, University of Lincoln, Brayford Pool, Lincoln LN6 7TS, UK; 4 Lincoln Medical School, College of Science, University of Nottingham and University of Lincoln, Lincoln LN6 7TS, UK; 5 La Trobe Rural Health School, College of Science, Health and Engineering, La Trobe University, Bendigo, VIC 3086, Australia

**Keywords:** cancer survivors, cancer survivorship, oncology, rapid review, systematic review, rural health, COVID-19, pandemic

## Abstract

The COVID-19 pandemic has caused considerable disruption to cancer care and may have exacerbated existing challenges already faced by cancer survivors from rural areas. This has created a need for a rapid evidence synthesis to inform the development of tailored interventions that address the specific needs of rural cancer survivors who continue to be affected by the pandemic. The review was conducted following guidance from the Cochrane Rapid Review Methods Group. Database searches were performed via the EBSCOHost interface (includes MEDLINE, CINAHL, PsycINFO) on 25 May 2022 and supplemented with searches on Google Scholar. Peer-reviewed articles published after March 2020 that reported primary data on the experiences of cancer survivors residing in rural and remote settings during the pandemic were included. Findings were tabulated and written up narratively. Fourteen studies were included. The COVID-19 pandemic had a mostly detrimental impact on the experiences of rural cancer survivors. People’s individual coping mechanisms were challenging for a range of reasons. Specifically, the pandemic impacted on their ability to access testing, treatment, check-ups and supportive care, their ability to maintain and access social support with close friends and family, as well as negative consequences to their finances and emotional wellbeing with some reporting feelings of psychological distress including depression and anxiety. This review provides important insight into the experiences of rural cancer survivors that may help inform tailored support in line with the needs and challenges faced because of the pandemic.

## 1. Introduction

There are over 17 million individuals living with cancer in North America [1] and an estimated 3 million in the UK, where cases are predicted to rise to nearly 5.3 million by 2040 [2], with similar patterns of increase projected throughout Europe [3]. Since the beginning of the COVID-19 pandemic, there has been considerable global disruption to the way cancer care has been delivered [4]. Screening and diagnostic services have been drastically reduced and treatment pathways were altered to mitigate the potential risk of COVID-19 exposure [5,6]. Key elements of ongoing care of existing patients were also deprioritised to allow health systems to better tackle the COVID-19 pandemic, resulting in the suboptimal delivery of cancer care [7]. Whilst healthcare systems have rapidly reorganised services to ensure patients continue to receive essential care, the pandemic has had a significant impact on cancer patients and survivors with respect to adapting to alternative healthcare delivery and managing their own physical, psychosocial, and economic wellbeing [8]. In sum, the pandemic has the potential to disproportionately affect and disrupt the lives of cancer survivors in the short, medium and long-term, including those currently receiving treatment, those who have completed treatment, and those who are now living disease free [9].

Prior to the COVID-19 pandemic it had been widely acknowledged that significant disparities already exist between cancer survivors who reside in urban and rural areas [10,11,12,13,14,15,16]. Individuals residing in rural and regional areas are shown to have higher cancer and other causes mortality in addition to an increased risk of experiencing worse health outcomes, poorer long-term survival, and greater unmet psychosocial needs compared to their urban counterparts [10,16,17,18,19,20,21]. This may be further exacerbated by additional challenges including greater travel distance to receive treatment, limited access to health and care facilities and to bespoke or individually tailored support [22]. Research also suggests that people with cancer from rural communities may be at risk of experiencing a reduction in quality of life following the completion of treatment [23], reflecting the need to provide tailored supportive care to improve their quality of life [24]. A recent systematic review concluded that rural survivors should be seen as having unique psychosocial needs when compared to urban survivors but not necessarily greater levels of need [17]. It is important to note that there are a range of characteristics belonging to rural communities that can positively influence the experiences of cancer survivors who reside in rural areas [25,26,27]. For example, people living in rural communities frequently report more close relationships with family and friends and sometimes have access to greater levels of community support and green spaces which can improve both physical and mental health [15,28,29,30]. Rural cancer survivors can have unique values and different attitudes to help-seeking as well as maintaining a stronger degree of stoicism [31]. Furthermore, rural cancer survivors have been shown to have greater cancer-related self-efficacy, engagement with self-management and higher self-reported health status when compared to their urban counterparts [30,32,33] and are less likely to report higher levels of distress [34].

As the COVID-19 pandemic continues to influence the way cancer care is delivered, it may also further exacerbate the unique set of existing challenges that cancer survivors from rural areas already face. A recent rapid review examined the broad impact of the pandemic on cancer survivors but did not report any findings in relation to rurality [35]. Furthermore, the review included studies published between Dec 2019–Aug 2020. There is therefore an urgent and timely need to conduct a more up to date evidence synthesis to help inform the development of tailored interventions that will address the specific needs of rural cancer survivors who continue to be affected by the pandemic. It is also important to note that there were stark geographic and regional differences regarding the impact of COVID-19 including between rural and urban areas [36]. Moreover, there were differences in the timing and duration of local lockdowns including public health imposed social isolation measures [37]. The aim of this research was to conduct a rapid systematic review of the peer-reviewed evidence to explore the impact of the COVID-19 pandemic on cancer survivors who reside in rural areas.

## 2. Methods

### 2.1. Protocol Registration

The review protocol has been registered on the Open Science Framework [https://osf.io/2fjgc/, accessed on 2 September 2022] to promote reproducibility and facilitate methodological transparency [38].

### 2.2. Study Design

The study utilised a rapid review approach in line with the published guidance from the Cochrane Rapid Reviews Methods Group [39] and for reporting used the Preferred Reporting Item for Systematic Reviews (PRISMA) checklist [40]. Rapid reviews are now considered a key component of the knowledge synthesis family alongside systematic reviews, scoping reviews, and realist reviews [41]. They provide a streamlined, efficient, and pragmatic approach to evidence synthesis. In summary, rapid reviews are a form of evidence synthesis in which components of the systematic review process are simplified, with a view to producing findings in a timely manner [42,43]. Still, rapid reviews must remain systematic in their approach and have a duty to report their methods in a transparent manner making sure they are clear about deviations or omissions from the PRISMA criteria.

### 2.3. Search Strategy

The search strategy was devised by DN, SC and BMcL. DN performed keyword searches together with Boolean operators (OR and AND) and truncation (*) to search for relevant peer-reviewed literature in EBSCOHost (includes MEDLINE, CINAHL and PsycINFO) on 25 May 2022. The EBSCOHost interface provides access to a range of academic databases in relation to health, allied health, nursing, social science, and multi-disciplinary literature. The syntax included keywords and alternative terms in relation to (1) cancer survivors (2) rurality and (3) COVID-19. The full search strategy for EBSCOHost can be found at Table 1 below. All database searches were supplemented by searches on Google Scholar to ascertain if there were any important literature that we missed from our initial search.

To identify cancer survivors the following keywords were used: “Cancer survivors” OR “Cancer survivorship” OR “Living with and beyond cancer” OR “Cancer patient” OR “People affected by cancer” OR “People living with cancer” OR “People living with and beyond cancer”

To identify rural settings the following keywords were used: “Rural health” OR “Rural health service*” OR Rural* OR Remote OR “Remote population” OR “Rural communit*” OR “Medically underserved area” OR Non-urban OR Nonurban OR Non-metropolitan OR “Geographic isolat*”

To identify evidence related to the COVID-19 pandemic the following keywords were used: COVID-19 or Coronavirus OR 2019-nCoV OR SARS-CoV-2 OR COV-19 OR “COVID-19 pandemic”

All retrieved records were collated into a data file and then stored and managed using Rayyan software [44]. The titles and abstracts were screened against the eligibility criteria by DN and AN. Where there were uncertainties over the eligibility of a study DN discussed this with SC to reach a decision. Following title and abstract screening, the remaining articles were independently screened by full text for inclusion by BMcL and then checked by DN and SC.

#### Eligibility Criteria

(1) Inclusion Criteria

Peer-reviewed publications were selected for inclusion in the review if they met the following pre-defined eligibility based on the PICO approach. Population: People who have (or who have had in the past) a confirmed diagnosis of cancer as well as people who support them (e.g., spouse, partner, children and health professionals). Intervention: Articles published after 11 March 2020 (beginning of COVID-19 pandemic and global lockdowns as identified by the World Health Organisation) that explore the experiences of cancer survivors and their caregivers (informal and formal) who reside in a rural, remote or regional area during the COVID-19 pandemic. Comparison: Not applicable. Outcomes: Articles that report primary data (quantitative and/or qualitative) on individual experiences, perceptions, reflections, beliefs, and attitudes of rural cancer survivors and the people who support them. Only publications written in the English language were included with no limitations set on geographical location

(2) Exclusion Criteria

Publications were excluded based on the following exclusion criteria. Population: People who do not have a confirmed diagnosis of cancer or people who do not directly support them. Intervention: Articles published prior to 11 March 2020 or published articles that do not explore the experiences of cancer survivors and their caregivers (informal and formal) who reside in a rural, remote or regional area during the COVID-19 pandemic. Comparison: Not applicable. Outcomes: Articles that do not report primary data on individual experiences, perceptions, reflection, beliefs, and attitudes of rural cancer survivors and the people who support them. Any publication that was not written in the English language were excluded.

### 2.4. Data Extraction

The first author (DN) led on data extraction for each full text article (see Table 2) with cross checking for accuracy taking place by the second author (SC). The following study characteristics were extracted including: (1) authors, title, year of publication and country (2) setting (3) participants (4) methods and (5) findings in relation to rurality.

### 2.5. Quality Assessment

The focus of this rapid review was on exploring the experiences of cancer survivors who reside in rural areas during the COVID-19 pandemic, therefore a quality assessment of included articles was not deemed appropriate. The omission of a quality assessment was in line with the methodological approach taken by other rapid systematic reviews where the focus is on producing evidence quickly [45].

### 2.6. Data Analysis

To identify and map the experiences of rural cancer survivors during the COVID-19 pandemic we tabulated the results. This was accompanied by a narrative summary where comments on the prominent issues within the literature were reported. Due to the wide heterogeneity of the design and outcomes of included studies, as well as the considerable amount of qualitative data, a formal statistical meta-analysis was not conducted. Furthermore, the need to produce findings quickly meant that a formal thematic synthesis was not possible.

**Table 2:** Characteristics of included studies.

Authors and Title	Setting	Participants	Methods/Design	Findings in Relation to Rurality
**Baffert et al. (2021)** [46] Quality of life in patients with cancer during the COVID-19 pandemic (France)	Patients managed in a day hospital of the Medical Oncology Department at Limoges Dupuytren Hospital which is located in a rural area.	*n* = 189 Patients mostly aged 61–70, female (*n* = 113) and male (*n* = 64) who presented with lung, breast or colorectal cancer.	Quantitative study design A prospective observational study on patient-reported outcomes, quality of life and satisfaction in patients with cancer with their care management in a day hospital during May–June 2020.	Rural *n* not reported but the authors refer to the hospital being in a rural setting in the French countryside and that many of the patients that go there reside in rural areas. Overall, patients’ anxiety scores remained low post-lockdown in rural regions where the rate of infection was limited and where patients had preserved quality of life which the authors infer could be by having a nice home in a rural area and where the organisation of care was globally maintained. Identified risk factors for post-lockdown anxiety were female gender and living in a city apartment.
**Davis et al. (2021)** [47] Exploring the challenges in accessing care and support for cancer survivors in Australia during COVID-19 (Australia)	Cancer community wellness center in rural/regional Australia. The center cares for cancer survivors and caregivers at all stages of the care trajectory.	*n* = 66 Cancer survivors with a range of diagnoses, 71% female and 29% male (*n* = 19), 6% 40 and under (*n* = 4), 42% 41–64 (*n* = 28) and 52% 65+ (*n* = 34).	Qualitative and quantitative study design Completed an online survey via Qualtrics regarding the impact of COVID-19 on their access to medical and support services. Data were collected July-October 2020.	Authors refer to total *n* as rural/regional cancer survivors (*n* = 66). **Quantitative data** Reduced their social support from family and friends (59%) Reduced ability to see their health care providers (46%) Impacted their access to supportive services (44%) Increased their distress over their cancer and health (40%) Negatively impacted their emotional wellbeing including depression and/or anxiety (35%) caused major worry in their life (33%) Delayed their testing or checkups for their cancer (20%), and delayed their access to cancer treatment (15%) Older adults reporting that COVID-19 had a significantly greater impact in their ability to access supportive services, their ability to obtain social support from family and friends, feeling distressed over cancer and health and their overall quality of life. **Qualitative data** Participants indicated social concerns including isolation from friends and family. Other concerns related to purchasing of groceries and social distancing. Some also reported concerns with access to treatment and the government’s ability to cope with the pandemic Most helpful during the pandemic was access to friends and family via online services or telephone. Access to online activities such as yoga, support services and cancer communities were also identified as helpful. Also extra time to engage in physical activity was helpful.
**Galica et al. (2021)** [48] Coping during COVID-19: a mixed methods study of older cancer survivors. (Canada)	Patients discharged from care of their cancer team at the Cancer Centre of South-eastern Ontario, Kingston, Ontario, Canada	*n* = 30 Older adults (>60 years) who were recently (<12 months) discharged from care of their cancer team	Mixed methods study design Quantitative data were collected using the Brief-COPE questionnaire. Qualitative data were collected using telephone interviews to explore experiences and strategies for coping with cancer-related concerns. No dates for data collection are given but the authors do report that this took place during the pandemic. The article was published in Jan 2021 so we can assume that data collection took place post March in 2020.	37% of participants resided in rural area (*n* = 11). Participants appreciated how the privileges of financial planning for retirement, downsizing/renovating their living space, preparing living wills/funeral, and their living circumstances (e.g., not in long term care, in a rural area, an independent retirement community) provided ‘insulation’ from the negative effects of COVID-19. However, for one disabled participant living in a rural area, her in-home supportive services ceased due to the pandemic, which she said: “made me very confined because everything in this country is based on driving a car. …I have to arrange for somebody to do the groceries… it has affected me in a big way.
**Himbert et al. (2022)** [49] Factors associated with changes in exercise behavuours during the COVID-19 pandemic. (USA)	Cancer patients enrolled in the Total Cancer Care study, Huntsman Cancer Institute, Utah, USA	*n*= 1210 Adult cancer patients (>18 years)	Quantitative study design COVID-19 questionnaire (demographics and changes in exercise habits, health behaviours and psychosocial factors) Zip codes were categorised as urban or rural using the Rural-urban commuting Area Codes (RUCA) classification system. Data were collected from August–September 2020.	27% of participants resided in rural areas (*n* = 328) Patients living in rural areas appeared not to change their exercise habits as compared to exercising less or more (32% vs. 21% or 19%; *p* < 0.0003). Higher percentage of urban patients reported changes in their exercise habits.
**Howden et al. (2021)** [50] A Cross-Sectional Survey Exploring the Impact of the COVID-19 Pandemic on the Cancer Care of Adolescents and Young Adults (Canada)	Participants were recruited across Canadian through an online survey link via cancer support group websites.	*n* = 805 Adolescents and young adults (18–39 years old) Diagnosed with any types of cancer	Quantitative and qualitative study design Data obtained through prior national cross-sectional survey. Survey created and administered online using REDCap. Data collected between January–February 2021.	22% of participants resided in rural areas (*n* = 179) and 2% in remote areas (*n* = 17). Province/territory of residence was associated with experiencing at least one negative impact on cancer care (*p < 0.03*). The odds of having a negative impact on cancer care were higher for participants living in Central Canada, the Prairies and British Columbia compared to those living in the Territories of Canada.
**Howden et al. (2022)** [51] Loneliness among adolescents and young adults with cancer during the COVID-19 pandemic: a cross-sectional study (Canada)	Participants were recruited across Canadian through an online survey link via cancer support group websites.	*n* = 805 Adolescents and young adults (18–39 years old) Diagnosed with any types of cancer	Quantitative study design. Data obtained through prior national cross-sectional survey. Survey created and administered online using REDCap. Loneliness was measured using the 3-item UCLA Loneliness Scale. Data collected between January–February 2021.	22% of participants were from rural (*n* = 179) and 2% from remote areas (*n* = 17). Participants who lived in rural or remote locations were less likely to experience loneliness (AOR 0.59, 95% CI 0.40–0.87, *p* = 0.008) than those living in urban settings. Authors comment that regional differences in the burden of COVID-19 cases, duration of lockdowns and degree of public health restrictions may have resulted in different social isolation restrictions in different locations in Canada. Also, sense of community and belonging can be more strongly developed in rural areas due to smaller population sizes.
**Krok-Schoen et al.** (2021) [52] Experiences of healthcare providers of older adults with cancer during the COVID-19 pandemic (Australia, Canada, Denmark, France, Germany, Hong Kong, Italy, Japan, Mexico, Netherlands, South Africa, Spain, UK, USA)	Respondents worked in academic/NCI comprehensive care program, hospitals, integrated network cancer programs, and other settings	*n* = 274 Healthcare providers of older adults with cancer. Social workers (43%), oncologists, geriatricians, advanced practice providers (28%), administrators (8.1%), navigators (5.1%), multiple professions or positions (6.3%), other professions (9.2%).	Qualitative study design Questionnaire survey data obtained from a larger study. Focus on analysing the qualitative findings. The survey was live from April–May 2020.	17% of respondents were located at a cancer programmes/institutes in a rural area (*n* = 47). A prominent theme identified included telehealth challenges. Telehealth challenges included access and support issues as well as communication difficulty due to sensory impairment, rurality, and inadequate equipment. Disparities in access was another reported telehealth challenge. One healthcare provider noted, “patient’s tech ability, no internet, no computer, or smart phone availability” while another stated, “rural settings lack internet service.”
**Peoples et al. (2022)** [53] Impact of the COVID-19 pandemic on rural and urban cancer patients’ experiences, health behaviors, and perceptions. (USA)	Across 3 Cancer Centers: University of Utah Huntsman Cancer Institute, University of Miami Sylvester Comprehensive Cancer Center and Moffit Cancer Center	*n*= 1472 Adult cancer patients with a range of different tumour sites.	Quantitative study design COVID-19 survey between August–September 2020 either electronically, in-person/via mail or over the phone. Assessed the impact of the pandemic on medical appointments, prevention/health behaviours and psychosocial factors.	27% were from rural areas (*n* = 397) Rural versus urban patients were more like to be older, not employed, uninsured, former/current smokers, consume alcohol and have pandemic-related changes/cancellations in surgery appointments. Urban versus rural patients were more likely to socially distance, use masks and hand sanitizer and experience changes in exercise habits and in their daily lives.
**Podubinski et al. (2021)** [54] Experience of Healthcare Access in Australia during the First Year of the COVID-19 Pandemic. (Australia)	Individuals living within Australia	*n* = 59 Individuals living within Australia who raised issues related to healthcare access in a wider COVID-19 study.	Qualitative study design Semi-structured interviews conducted between August–December 2020 via phone or video conference. Participants were selected from a larger pool of respondents who had completed an online, nationally distributed survey on transmission and compliance with isolation, hygiene and social distancing measures.	Delay in access to specialist care was a major issue raised by people who lived away from major metropolitan areas, and the effect was cumulative across multiple types of care, particularly for older people. “We’ve put off going to specialists… its two and a half hours away from us. Both my husband and I have skin cancers that have to be removed, which we haven’t had done… and optometrists and dental appointments. Yeah. So those sorts of appointments have had to be cancelled. Because we can’t travel.”
**Rajan et al. (2021)** [55] Impact of COVID-19 pandemic on cancer surgery: Patient’s perspective (India)	The study was conducted in the Department of Surgical Oncology, at a tertiary care referral centre.	*n* = 310 Cancer patients (>18 years), male (*n* = 187, 60%) and female (*n* = 123, 40%)	Quantitative study design A cross-sectional survey that collected data on finances, access to healthcare, anxiety, stress and depression. Data were collected between June–August 2020.	73% of participants resided in rural areas (*n* = 227) Patients who belonged to rural areas were impacted significantly more than those living in urban areas in terms of healthcare access (mean difference, 35 vs. 31, *p* = 0.015). The hospital is located in an urban area and as urban people had better access to transport, they were less impacted in accessing healthcare. Higher odds of COVID-19 related impact in patients from rural areas. Difficulty in reaching the hospital and arranging accommodation due to the majority of the sample coming from rural areas.
**Ratnapradipa et al. (2022)** [56] Qualitative analysis of cancer care experiences among rural cancer survivors and caregivers (USA)	Rural Cancer Centers located in central and western Nebraska	*n* = 20 Cancer survivors/patients (*n* = 16) and caregivers (*n* = 5). Female (*n* = 16) and Male (*n* = 4). Breast (*n* = 2), Bladder (*n* = 1), Multiple myeloma (*n* = 2), Lymphoma (*n* = 2), Prostate (*n* = 1)	Qualitative study design. Three 75–90 min. focus groups were conducted between Feb–May 2021 with 6–8 participants per group. Focus group questions were based on a community health needs assessment and the extant literature. Questions were asked in relation to community health status, cancer experiences (diagnosis and treatment, sources of cancer-related information).	The authors refer to the total *n* as being in a rural setting (*n* = 20) Participants identified barriers to care, including finances (treatment costs/insurance), transportation and lack of support groups and social support, as well as, more general cultural barriers. Participants noted the challenges with rural cancer centres retaining specialists referring to the turnover as “radiation oncologist of the month”
**Singh et al. (2021)** [57] Impact of COVID-19 lockdown on patients with cancer in North Bihar, India: A phone-based survey. (India)	Preventive oncology clinic in the Muzaffarpur district of North Bihar, India	*n* = 210 Cancer patients, majority were women (63%) and aged more than 40 years (77%).	Quantitative study design Descriptive, cross-sectional telephone survey. Data were collected between April–May 2020. Questions related to the patients characteristics, type of cancer, their current and past treatment status, impact of the lockdown on their follow-up appointments, treatment, and surgery, or any other problems they may have faced during the lockdown that affected their treatment.	60% of participants resided in rural areas (*n* = 125) Most patients who missed their scheduled appointments were aged more than 60 years, women, inhabitants of rural areas, with multiple comorbidities, or belonged to the lower middle-income economic strata. About 70% of the patients faced transportation issues, and 55% experienced financial problems during the lockdown.
**Spencer-Bowdage et al. (2021)** [58] The experience of UK patients with bladder cancer during the COVID-19 pandemic: a survey-based snapshot (UK)	Action Bladder Cancer UK website, Patient Support Groups and Social Media Platforms. Geographical reach included from all the UK	*n* = 156 Patients with bladder cancer	Quantitative study design Online questionnaire survey. Data were collected between April–July 2020.	34% of participants were from rural areas (*n* = 53). Our survey appears to demonstrate that both MIBC and NMIBC patients have been equally affected by delays, postponements and cancellations during the COVID-19 pandemic. There was no association between area type (rural/urban) and disruption to cancer treatment during the COVID-19 pandemic.
**Zomerdijk et al. (2021)** [59] Prevalence and correlates of psychological distress, unmet supportive care needs, and fear of cancer recurrence among haematological cancer patients during the COVID-19 pandemic. (Australia)	Online advertisement distributed via haematology groups. Geographic reach included from all of Australia.	*n* = 394 Patients with haematological cancer diagnosis	Quantitative study design An online cross-sectional survey was conducted that explored wellbeing, psychological distress, unmet supportive care needs and fear of cancer recurrence. Data were collected between July–August 2020.	50% of participants lived in regional areas (*n* = 196). Living in a ‘regional’ area were associated with greater psychological distress during the pandemic.

## 3. Results

### 3.1. Search Results

**Figure 1:**
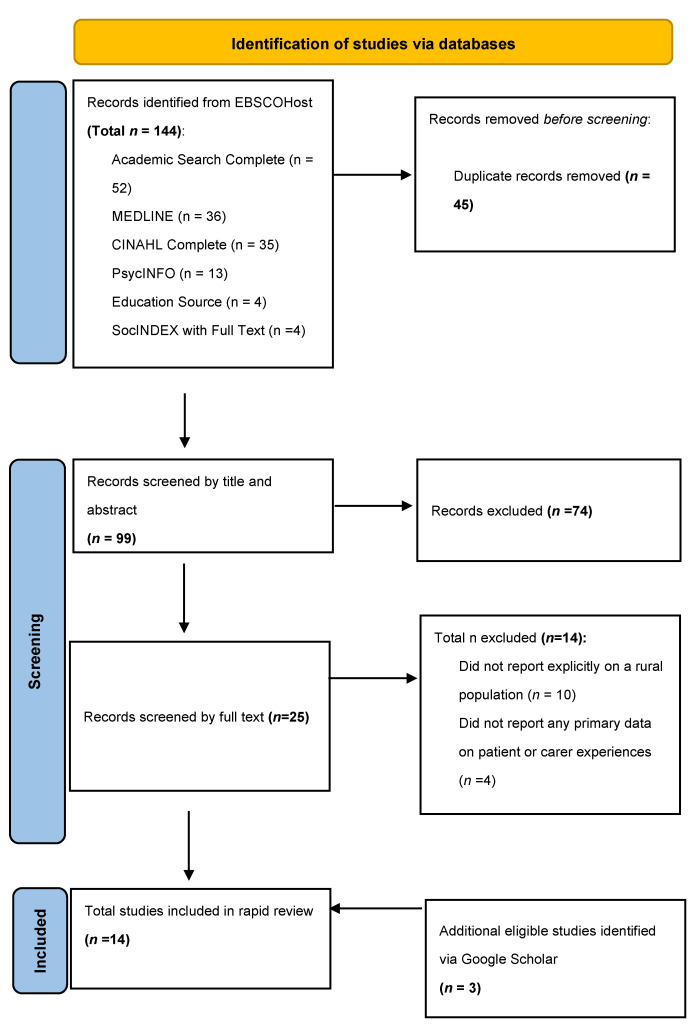
PRISMA Flow Diagram.

The search of EBSCOhost provided a total of 144 citations. Of these 144, 45 were duplicates and were excluded leaving 99 records that were then screened by title and abstract. Following title and abstract screening a further 74 articles were excluded leaving 25 to be screened by full text. Fourteen articles did not meet the eligibility criteria following full text screening. The primary reason was that the article did not explicitly report on whether all or some of their participants were resident in a rural area. A total of three additional articles were identified via Google Scholar leaving a total of twelve articles that met the pre-defined eligibility criteria were included in the final review. The process has been reported on in the PRISMA Flow diagram below (see Figure 1).

### 3.2. Study Characteristics

A total of fourteen studies were included in the review [46,47,48,49,50,51,52,53,54,55,56,57,58,59]. The studies were conducted in a range of different geographies in mostly high income countries and to a lesser extent low-middle income countries: Australia *n* = 3 [47,54,59]; Canada *n* = 3 [48,50,51]; France *n* = 1 [46]; India *n* = 2 [55,57]; United Kingdom *n* = 1 [58]; United States of America *n* = 3 [49,53,56]; Mixed geographies *n* = 1 [52]. In studies with both rural and non-rural participants the rural/remote/regional *n* ranged from seventeen to seventy-three per cent of the total sample [48,49,50,51,52,53,55,57,58,59]. Only two studies reported their total *n* as coming from rural or regional areas [47,56]. Baffert et al. [46] did not report how many of their participants were resident in a rural area but did refer to the hospital being located in a rural setting and report on some of their findings unique to rural participants as well as the differences they found between those in rural versus city locations.

Most studies collected data with cancer survivors with a range of different diagnoses [46,47,48,49,50,51,53,55,56,57]. Only one study (Krok-Schoen et al.) looked explicitly at healthcare providers as opposed to cancer survivors [52]. One UK study looked specifically at patients with bladder cancer [58] and another Australian study collected data with people who had a haematological cancer diagnosis [59]. The Australian study by Podubinski et al. [54] did not focus specifically on cancer survivors, rather they looked at individuals in Australia who raised issues around healthcare access in a COVID-19 study, they do report some primary qualitative experience data with people who had skin cancer and were resident in rural areas therefore their article was included. Only one study explicitly reported on and included caregivers in their sample [56].

Most of the studies (*n* = 8) reported using a solely quantitative design [46,49,51,53,55,57,58,59]. Two studies reported collecting both quantitative and qualitative data [47,50] but only one study explicitly reported adhering to a mixed methods design [48]. Finally, three studies used only qualitative methods to collect their data [52,54,56]. Across all the studies data were collected between April 2020–May 2021 [46,47,49,50,51,52,53,54,55,56,57,58,59] with the exception of research by Galica et al. [48] which did not report the data collection period but did report that this took place during the COVID-19 pandemic. The characteristics of the included studies is reported on in Table 2.

### 3.3. Narrative Overview

Across the included studies, the COVID-19 pandemic had a mostly detrimental impact on the experiences of rural cancer survivors. People’s individual coping mechanisms were challenging for a range of reasons. Specifically, the pandemic impacted on their ability to access testing, treatment, check-ups and supportive care, their ability to maintain and access social support with close friends and family, as well as negative consequences to their finances and emotional wellbeing with some reporting feelings of psychological distress including depression and anxiety [47,48,52,53,54,55,56,57,58,59]. In an Australian study with a solely rural/regional sample (*n* = 66), older participants (65 years and over) were more negatively impacted by the pandemic with their ability to access supportive services and to access social support when compared to younger participants [47]. The same study found no significant differences between gender or cancer status and access to services [47]. In America, rural cancer survivors were more likely to be older, unemployed, uninsured, former or current smokers, to consume more alcohol and suffer from pandemic related changes to their medical appointments compared to those from urban areas [53]. Other American research with cancer survivors and their caregivers highlighted the workforce challenges around recruiting and retaining specialist Cancer Center staff in rural areas in Nebraska [56].

In Australia, living in a regional area, was associated with greater psychological distress during the pandemic [59]. Although in France, cancer patients’ anxiety scores remained low post-lockdown in rural regions where the rate and risk of COVID-19 infection was lower and the organisation of care was considered to be mostly maintained [46]. Furthermore, they found that living in a city apartment was an identified risk factor for post-lockdown anxiety. The odds of having a negative impact on cancer care were higher for participants living in Central Canada as opposed to those living in the Territories which are much more rural and remote [50]. Those living in rural or remote locations in Canada were less likely to experience loneliness than those from urban areas which could be down to a strong sense of community and belonging in these rural areas with smaller population sizes [51]. Although there were reports of the need to have access to a car as well as support with grocery shopping for those that did not own a vehicle. American research found that urban cancer survivors were more likely to socially distance, wear a face mask and use hand sanitizer as well as experience significant changes in their daily lives when compared to those from rural areas [53].

The only UK study that was included in the review found that the level of disruption between rural and urban participants was not statistically significant and that both sets of patients had been equally impacted by delays and cancellations to treatment during the COVID-19 pandemic [58]. The two studies from India that were included found that those from rural areas were negatively impacted in terms of access and that urban people had better access to transport and consequently were less impacted in accessing their care [55,57]. People from rural India were more likely to miss their scheduled appointments [57] due to difficult in reaching the hospital and arranging accommodation [55].

The use of the telephone and online services to connect with friends and family was considered a helpful strategy for staying in touch where physical contact was not possible [47]. Online activities extended beyond keeping in touch with friends and family to include things such as yoga and cancer support groups [47]. The increased use of telehealth and digital technologies were not without their challenges and these included access and support issues as well as communication and connectivity difficulties due to sensory impairment, rurality and inadequate equipment [52]. There was a perception that living in a rural area with a lower population density provided some degree of ‘insulation’ from the negative effects of COVID-19 [48] and some rural cancer survivors in Australia reported that there were now more opportunities to engage in physical activity practices compared to before the pandemic [47]. In the USA, patients living in rural areas appeared to not change their exercise habits as much when compared to their urban counterparts [49,53].

## 4. Discussion

This is the first review to report solely on the experiences of rural cancer survivors during the COVID-19 pandemic. Rapid reviews are increasingly becoming a well-established methodology to produce findings quickly in relation to topical and important research areas [41] and this review builds on a recently published rapid review on the broader impact of COVID-19 on cancer survivors [35]. The searches for that review were conducted in August 2020 and our search in comparison was completed almost two years later in May 2022 as well as including a more developed search strategy with the addition of terms in relation to rurality. Therefore, these findings provide new and more up-to-date findings for cancer researchers, specifically those with an explicit interest in cancer care and rurality.

This research was further strengthened by a predetermined study protocol and adherence to the PRIMSA guidelines ensuring that the process was systematic and transparent [39,40]. Another strength of this review was the geographic reach of the articles with the inclusion of literature from a range of different countries in both high and low-middle income settings. Not surprisingly, the majority of the research that was included in this review was conducted in North America and Australia which is similar to pre-pandemic scholarship on rurality and cancer that has been dominated by these areas for the past decade [11,12,13,15,16,17,18,31,34]. Only two of the COVID-19 studies that were included in this rapid review were from the European continent with one of these being from the UK where there is now a small but increasing body of pre-pandemic research that considers the impact of rurality on cancer experiences and outcomes [30,32,33]. Future research that looks at cancer and geographic residence, particularly that in the UK, should extend out to include coastal areas which are amongst the most deprived in the country and there is now a shifting policy focus to reduce growing health inequalities in deprived coastal communities [60].

It has been acknowledged that the challenges presented by the COVID-19 pandemic may disproportionately impact the overall health and wellbeing of cancer survivors which can result in unintended long-term consequences [35] and it could be argued that those who reside in rural and remote areas are particularly susceptible to disparities in cancer care and poorer health outcomes [61]. Indeed, this review has provided novel insight into the experiences of cancer survivors who resided in rural areas during the pandemic and has highlighted the negative impact on their ability to access care and social support as well as on their mental health. Social support has been identified as being beneficial to the management of depressive symptoms [62] and not being able to access it could have considerable negative consequences for rural cancer survivors’ mental health. It is important to acknowledge pre-COVID-19 research that suggests distance might not always be such a salient concern and that rural dwellers sometimes accept their issues around access given that some of them have lived in a rural area for a prolonged period of time [63]. Telemedicine is a scalable technology that has largely been a response to the issues and needs of rural providers and people [64]. The use of telemedicine approaches is certainly not novel and has been used to facilitate access to care for people in rural, remote, and non-rural settings for several years now. During the initial stages of the pandemic, healthcare providers including oncology nurses across the world were required to rapidly shift to a telehealth model of delivery to provide care to people living with cancer [65,66,67]. Whilst this has considerably increased engagement with digital healthcare, it should not be considered a total solution for the healthcare challenges in rural areas. There is a need to consider digital health literacy and digital poverty particularly in rural areas with high levels of deprivation [68]. This review has shown that access to digital technology and connectivity issues are still prominent within rural settings and so investment as well as education is required [69] to ensure that digital inequalities do not widen as telemedicine models of care continue to be rolled out in a post-pandemic world. People in rural areas may also rely on digital health information provided through social media platforms such as YouTube although the extent to which these provide good quality and reliable information has been questioned by other cancer researchers [70,71].

For rural people, the literature suggests that ‘good health’ is commonly characterised as being able to work, engage in social relationships and to maintain independence [72]. Future interventions and support should be tailored to the needs of rural cancer survivors in relation to the medium and longer-term impacts of the pandemic as well as more generally. Importantly, the role and needs of informal caregivers should not be neglected especially in rural areas where cancer survivors can already lack access to both medical and social support [64]. Much like rural cancer survivors, informal and unpaid caregivers from rural areas are susceptible to high levels of depression and anxiety, feelings of isolation and financial problems because of their role as a carer [31,73,74]. Despite being part of the inclusion criteria for this review, there was only one study that looked explicitly at the experiences of informal caregivers of cancer survivors in rural areas during COVID-19 and future research should make concentrated efforts to include and involve them. Whilst evidence from this review and the wider literature continues to highlight a workforce shortage of both health and mental health professionals in rural and remote areas [64,75], informal carers and the wider community have a vital role to play in supporting people through their diagnosis, treatment, and recovery.

Much like the wider health sciences literature, the evidence base within cancer research is often weakened by the absence of good quality studies with parallel comparison groups [72]. This would allow us to verify and or challenges some of the negative and positive assumptions in relation to health and rural-urban residence. In order to ascertain the longer-term fallout of the pandemic on the experiences of rural cancer survivors future primary research and good quality evidence syntheses will be warranted.

### Limitations

There are several limitations to this review. Whilst it followed a systematic and comprehensive search strategy using a pre-defined protocol, this was not a full systematic review and as such there could be studies that were missed via the search strategy detailed above. Future searches should be optimised by searching additional multi-disciplinary databases such as Scopus and Web of Science. The searching of reference lists and citations would also be welcomed in subsequent reviews of the literature. The omission of a quality assessment stage in the review is largely in keeping with the aims of a rapid review, however, we encourage future evidence syntheses in this area to consider evaluating the scientific quality of included studies so the academic community can be informed on the quality, importance and originality of the extant literature. Whilst we have noted that the inclusion of a wide range of studies from different geographic settings was a strength, the heterogeneity of settings also somewhat limits the local applicability of the findings where definitions and conceptualisations of ‘rural’ will greatly vary, as well as the public health and policy approaches to the management of COVID-19. Finally, future evidence syntheses will be warranted in the near future to ensure that all of the pertinent evidence from the COVID-19 pandemic is collated and available to the academic community who are interested in cancer and rurality. This rapid review could provide a useful template to inform the development of a full systematic review.

## 5. Conclusions

As we continue to deal with the longer-term impact of the COVID-19 pandemic, this review provides novel and important insight into the experiences of rural cancer survivors that may help inform tailored support in line with the additional needs and challenges they face because of the pandemic.

## Figures and Tables

**Table 1:** Search Strategy for EBSCOHost.

Search Terms	*N*
**Cancer Terms**	
“Cancer survivors”	176,739
“Cancer survivorship”	11,372
“Living with and beyond cancer”	376
“Cancer patient”	155,316
“People affected by cancer”	708
“People living with cancer”	846
“People living with and beyond cancer”	180
*“Cancer survivors” OR “Cancer survivorship” OR “Living with and beyond cancer” OR “Cancer patient” OR “People affected by cancer” OR “People living with cancer” OR “People living with and beyond cancer”*	**220,420**
**Rural Terms**	
“Rural health”	102,640
“Rural health service*”	29,762
Rural*	1,384,440
Remote	871,244
“Rural population”	120,872
“Rural communit*”	95,636
“Medically underserved area”	12,659
Non-urban	3986
Nonurban	2266
Non-metropolitan	4722
“Geographic isolat*”	4315
“Rural health” OR “Rural health service*” OR Rural* OR Remote OR “Rural population” OR “Rural communit*” OR “Medically underserved area” OR Non-urban OR Nonurban OR Non-metropolitan OR “Geographic isolat*”	**2,226,458**
**COVID-19 Terms**	
COVID-19	1,077,712
Coronavirus	790,904
2019-nCoV	255,918
SARS-CoV-2	255,990
COV-19	1,077,712
“COVID-19 pandemic”	506,126
*COVID-19 OR Coronavirus OR 2019-nCoV OR SARS-CoV-2 OR COV-19 OR “COVID-19 pandemic”*	**1,255,900**
**Combine above (1) Cancer terms (2) Rural terms and (3) COVID-19 terms with AND**	**144**
“Cancer survivors” OR “Cancer survivorship” OR “Living with and beyond cancer” OR “Cancer patient” OR “People affected by cancer” OR “People living with cancer” OR “People living with and beyond cancer” AND “Rural health” OR “Rural health service*” OR Rural* OR Remote OR “Rural population” OR “Rural communit*” OR “Medically underserved area” OR Non-urban OR Nonurban OR Non-metropolitan OR “Geographic isolat*” AND COVID-19 OR Coronavirus OR 2019-nCoV OR SARS-CoV-2 OR COV-19 OR “COVID-19 pandemic”	

Note: Search conducted on 25 May 2022.
